# Impact of Integrase Sequences from HIV-1 Subtypes A6/A1 on the *In Vitro* Potency of Cabotegravir or Rilpivirine

**DOI:** 10.1128/aac.01702-21

**Published:** 2022-03-15

**Authors:** Jerry L. Jeffrey, Marty St. Clair, Ping Wang, Chunfu Wang, Zhufang Li, Jagadish Beloor, Christine Talarico, Robert Fridell, Mark Krystal, C. Thomas White, Sandy Griffith, Ronald D’Amico, Kimberly Smith, Veerle Van Eygen, Johan Vingerhoets, Kati Vandermeulen, William Spreen, Jan van Lunzen

**Affiliations:** a ViiV Healthcare, Research Triangle Park, North Carolina, USA; b ViiV Healthcare, Branford, Connecticut, USA; c Janssen Pharmaceutica NV, Beerse, Belgium; d ViiV Healthcare, Brentford, United Kingdom

**Keywords:** resistance mutation, HIV-1 infection, integrase inhibitor, non-nucleoside reverse transcriptase inhibitor, long-acting, antiretroviral

## Abstract

The FLAIR study demonstrated noninferiority of monthly long-acting cabotegravir + rilpivirine versus daily oral dolutegravir/abacavir/lamivudine for maintaining virologic suppression. Three participants who received long-acting therapy had confirmed virologic failure (CVF) at Week 48, and all had HIV-1 that was originally classified as subtype A1 and contained the baseline integrase polymorphism L74I; updated classification algorithms reclassified all 3 as HIV-1 subtype A6. Retrospectively, the impact of L74I on *in vitro* sensitivity and durability of response to cabotegravir in HIV-1 subtype B and A6 backgrounds was studied. Site-directed L74I and mutations observed in participants with CVF were generated in HIV-1 subtype B and a consensus integrase derived from 3 subtype A6 CVF baseline sequences. Rilpivirine susceptibility was assessed in HIV-1 subtype B and A1 containing reverse transcriptase mutations observed in participants with CVF. HIV-1 subtype B L74I and L74I/G140R mutants and HIV-1 subtype A6 I74L and I74/G140R mutants remained susceptible to cabotegravir; L74I/Q148R double mutants exhibited reduced susceptibility in HIV-1 subtypes B and A6 (half maximal effective capacity fold change, 4.4 and 4.1, respectively). Reduced rilpivirine susceptibility was observed across HIV-1 subtypes B and A1 with resistance-associated mutations K101E or E138K (half maximal effective capacity fold change, 2.21 to 3.09). In cabotegravir breakthrough experiments, time to breakthrough was similar between L74 and I74 viruses across HIV-1 subtypes B and A6; Q148R was selected at low cabotegravir concentrations. Therefore, the L74I integrase polymorphism did not differentially impact *in vitro* sensitivity to cabotegravir across HIV-1 subtype B and A6 integrase genes (ClinicalTrials.gov identifier: NCT02938520).

## INTRODUCTION

Combination antiretroviral therapy (ART) is highly effective at achieving and maintaining viral suppression and prolonging life expectancy in people living with HIV (1, 2). Because of their increased efficacy and tolerability versus other ART drug classes, next-generation integrase strand transfer inhibitors (INSTIs) are recommended for use in first-line combination ART regimens and are the preferred first-line treatment option in several HIV-1 treatment guidelines (3–7). Although first-generation INSTIs raltegravir and elvitegravir demonstrated high efficacy (8–10), they have a relatively low genetic barrier to resistance (3). In contrast, second-generation INSTIs dolutegravir and bictegravir are effective (11–18), exhibiting improved resistance and durability profiles versus first-generation INSTIs (19). Treatment-emergent resistance to dolutegravir or bictegravir is rarely observed in patients naive to ART (20, 21). Furthermore, adults who were ART experienced but not virologically suppressed and had prior resistance to ≥2 ART drug classes had significantly less treatment-emergent INSTI resistance with dolutegravir versus raltegravir (18). These successful outcomes in part drove development of simplified INSTI-based regimens with a reduced pill burden, including novel, long-acting (LA) injectable formulations (3).

Cabotegravir, a structural analog of dolutegravir, is a second-generation INSTI with potent *in vitro* antiviral activity and a prolonged absorption rate–dependent half-life when administered as an intramuscular injection (22). Given its specific physiochemical and pharmacokinetic properties, cabotegravir was developed for use both as combination HIV-1 treatment and as monotherapy for HIV-1 prevention (22). For treatment, cabotegravir is approved in combination with the non-nucleoside reverse transcriptase inhibitor (NNRTI) rilpivirine as a complete LA regimen administered monthly (United States, Canada, Europe, and Australia) or every 2 months (Europe and Australia) (23–27).

The phase III FLAIR study evaluated the efficacy of monthly cabotegravir LA + rilpivirine LA in maintaining virologic suppression in adults with HIV-1 infection (ClinicalTrials.gov identifier: NCT02938520) (28). At Week 48, HIV-1 RNA ≥50 copies/mL was observed in 6 participants (2.1%) administered LA therapy versus 7 participants (2.5%) administered daily oral dolutegravir/abacavir/lamivudine (adjusted difference, −0.4%; 95% confidence interval [CI]: −2.8 to 2.1), demonstrating noninferiority of cabotegravir LA + rilpivirine LA versus oral dolutegravir/abacavir/lamivudine (28). Of 283 FLAIR study participants who received LA therapy, 3 participants (1%) had confirmed virologic failure (CVF), defined as 2 consecutive plasma HIV-1 RNA levels ≥200 copies/mL. All 3 participants had treatment-emergent INSTI resistance mutations of either G140R (*n* = 1) or Q148R (*n* = 2) and exhibited >5-fold reduced susceptibility to cabotegravir versus the reference virus. In addition, each participant had treatment-emergent NNRTI resistance mutations E138K, E138E/A/K/T, or K101E (*n* = 1 each), with the latter 2 participants showing >2-fold reduced susceptibility to rilpivirine versus the reference virus. All 3 participants with CVF were from Russia and had the integrase polymorphism L74I at baseline (28, 29). Furthermore, all 3 participants with CVF had the HIV-1 subtype that was previously reported as A1 (28).

The L74I polymorphism occurs in only 7% (404 of 5754) of integrase genes from isolates in the current Los Alamos National Laboratory (LANL) database of HIV sequences (http://www.hiv.lanl.gov/). L74I is particularly prevalent in individuals from Russia and former Soviet Union countries, where it is present in 93% to 100% of HIV-1 subtype A isolates (30). In the LANL database, L74I is present in only 5% (8 of 149) of integrase genes in HIV-1 subtype A1 isolates but is present in 92% (70 of 76) of integrase genes in HIV-1 subtype A6 isolates (http://www.hiv.lanl.gov/). Although L74I has not been observed in association with INSTI resistance (https://hivdb.stanford.edu/dr-summary/resistance-notes/INSTI/), the L74M mutation in combination with INSTI resistance-associated mutations (RAMs) T66I/K, E92V, Y143C, or N155H reduced susceptibility 14- to >200-fold to raltegravir or elvitegravir (31–34). L74M in combination with integrase mutations at positions 140 and 148 reduced susceptibility 6- to 12-fold to bictegravir, 10- to 12-fold to dolutegravir, and 53- to 220-fold to cabotegravir (35). Furthermore, L74M in combination with E92Q was found in an individual with CVF after raltegravir treatment (36). L74M alone has minimal impact on INSTI resistance (31, 37).

Given the reported INSTI resistance associated with the L74 position and the clustering of CVF among participants with the HIV-1 subtype that was previously reported as A1 and the L74I polymorphism in FLAIR, the possibility existed of a potential association between L74I and CVF, although the majority of participants with the baseline L74I polymorphism maintained virologic suppression at Week 48 (50/54; 93%) (28; https://hivdb.stanford.edu/dr-summary/resistance-notes/INSTI/). In addition, a recent *post hoc* multivariable analysis across the FLAIR, ATLAS, and ATLAS-2M phase III studies found that a combination of ≥2 baseline factors, including the presence of HIV-1 subtypes A6/A1 or baseline rilpivirine RAMs, was required to increase risk of CVF (28, 38–40). In this study, the relationship between L74I and HIV-1 subtype on sensitivity to cabotegravir and rilpivirine as well as the durability of response to cabotegravir was probed. An updated classification algorithm reclassified the HIV-1 subtype identified in the FLAIR participants with CVF as subtype A6, which is not surprising given the prevalence of L74I in HIV-1 subtype A6 viruses from individuals in Russia. In addition, the ability of cabotegravir to suppress replication of viruses of HIV-1 subtypes B and A6 with or without L74I was evaluated. Results from this study provide valuable insights into the pathway of HIV-1 resistance to cabotegravir and rilpivirine.

## RESULTS

### HIV-1 subtype reclassification.

All 3 FLAIR study participants receiving LA therapy with CVF were initially reported as having HIV-1 subtype A1 based on a commercial genotyping algorithm that only used protease (PR) and reverse transcriptase (RT) sequences (28). Given that the FLAIR participants with CVF presented with integrase mutations and cabotegravir resistance (28), their HIV-1 subtype was further evaluated with integrase sequences using the LANL reference library that contained additional HIV-1 subtypes, including A3, A4, and A6. The updated genotyping algorithm determined that viruses from the FLAIR study participants with CVF were most similar to HIV-1 subtype A6, rather than HIV-1 subtype A1 as originally reported (28). This is consistent with the high prevalence of L74I in HIV-1 subtype A6 (http://www.hiv.lanl.gov/).

### Cabotegravir sensitivity *in vitro*.

Sensitivity to cabotegravir (fold change in half-maximal effective concentration [EC_50_] values) was measured using replication-defective pseudoviruses to evaluate the impact of the L74I polymorphism in combination with integrase resistance mutations and HIV-1 subtype (Table 1). L74- and I74-containing NL_4-3_ (HIV-1 subtype B) viruses and chimeric NL_4-3_ viruses containing the ConA6 HIV-1 subtype A6 integrase sequence were examined for susceptibility to cabotegravir, and in the absence of other mutations, changes in integrase position 74 did not substantially affect EC_50_ values, with fold changes of 0.8 or 1.2 for the variants versus the parent viruses. These results suggest that L74I alone does not impact cabotegravir susceptibility in the context of NL_4-3_, an HIV-1 subtype B virus, or A6 integrase genes.

**TABLE 1:** *In vitro* cabotegravir inhibition of HIV-1 with integrase mutations^*a*^

Viral vector (HIV-1 subtype)	Wild-type	Site-directed mutant			
	Position 74	Position 74	Other	EC_50_, fold change^*b*^ (95% CI)	Adjusted *P* value
NL_4-3_ (B)	L74^*c*^	—	—	—	—
		L74I	—	1.2 (1.0, 1.6)	0.1948
		L74I	G140R	0.9 (0.7, 1.1)	0.4078
		L74I	Q148R	4.4 (3.5, 5.5)	<0.0001
ConA6 (A6)	I74^*d*^	—	—	—	—
		I74L	—	0.8 (0.6, 1.0)	0.1657
		I74	G140R	0.9 (0.7, 1.1)	0.6000
		I74	Q148R	4.1 (3.2, 5.3)	<0.0001

a ConA6, consensus A6; EC_50_, half maximal effective concentration.

b Fold change to respective wild-type virus in 3 independent experiments.

c Wild-type virus is a standard laboratory strain.

d Wild-type virus is derived from consensus integrase sequences obtained from the 3 viruses from FLAIR participants with confirmed virologic failure.

In addition to the L74I polymorphism, each participant who met CVF criteria had treatment-emergent INSTI resistance mutations of either G140R (*n* = 1) or Q148R (*n* = 2) (28). These additional mutations were also introduced into the clones to examine their effect on cabotegravir sensitivity. When G140R was introduced into the NL_4-3_ variant containing L74I and the ConA6 integrase-containing virus with I74, no change was observed in cabotegravir susceptibility, each with fold changes of 0.9 (Table 1). However, when Q148R was introduced into either clone, EC_50_ values against cabotegravir were increased, with fold changes of 4.4 and 4.1 for NL_4-3_ and ConA6-containing viruses, respectively. Similar results were observed for NL_4-3_ viruses with L74 and ConA6-containing viruses with I74L plus either G140R or Q148R integrase mutations (data not shown); these results are consistent with a previous study demonstrating that Q148R alone was sufficient to produce a 5-fold shift in EC_50_ values (41). Therefore, the L74I integrase polymorphism alone or in combination with Q148R did not differentially impact *in vitro* sensitivity to cabotegravir across these HIV-1 subtype B and A6 integrase genes.

### Rilpivirine sensitivity *in vitro*.

Sensitivity to rilpivirine (fold change in EC_50_ values) was measured by Monogram Biosciences to evaluate the impact of HIV-1 subtype in combination with the NNRTI RAMs observed in the 3 participants with CVF from FLAIR (Table 2). As the reclassification from HIV-1 subtype A1 to A6 was not known when the rilpivirine experiments were initiated, a reference strain of HIV-1 subtype A1 was selected to assess rilpivirine susceptibility. Wild-type NL_4-3_ (HIV-1 subtype B) virus and a chimeric NL_4-3_ virus with the PR/RT gene derived from an HIV-1 subtype A1 virus (92UG037) both had low fold change in EC_50_, with values of 0.89 and 0.71, respectively. Introduction of E138A or E138T into either clone resulted in fold change in EC_50_ values against rilpivirine of ≤2.0, the biological cutoff for rilpivirine. However, when E138K was introduced into either clone, EC_50_ values against rilpivirine were above the biological cutoff, with fold change values of 2.38 and 2.21 for the viruses with PR/RT genes from NL_4-3_ and 92UG307, respectively. Introduction of K101E also resulted in increased EC_50_ values against rilpivirine, with fold changes of 3.09 and 2.29 in the NL_4-3_ and 92UG307-PR/RT-containing viruses, respectively. Therefore, the known E138K and K101E mutations conferred EC_50_ fold change values consistent with *in vitro* resistance to rilpivirine equally across clones containing PR/RT genes from HIV-1 subtypes B and A1 (https://hivdb.stanford.edu/dr-summary/resistance-notes/NNRTI/).

**TABLE 2:** *In vitro* rilpivirine inhibition of HIV-1 with reverse transcriptase mutations^*a*^

	EC_50_, fold change^*b*^	
Mutation	NL_4-3_ (B)	92UG037 (A1)
WT	0.89	0.71
K101E	3.09	2.29
E138A	2.00	1.60
E138K	2.38	2.21
E138T	0.87	0.73

a EC_50_, half maximal effective concentration; WT, wild type.

b Fold change to reference virus in 3 independent experiments.

### Growth kinetics of replication-competent viruses.

**FIG 1:**
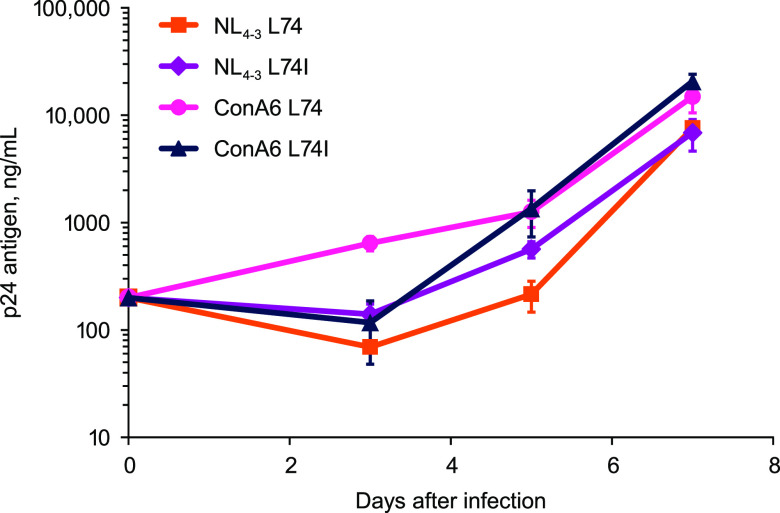
Viral replication kinetics of replication-competent NL_4-3_ and ConA6 viruses not containing luciferase reporters with and without integrase mutations at position 74 without cabotegravir treatment. Error bars indicate standard deviation. ConA6, consensus A6.

Replication-competent NL_4-3_ viruses with L74 or L74I and chimeric NL_4-3_ viruses containing ConA6 HIV-1 subtype A6 I74 or I74L integrase sequences were generated and used to infect MT-2 cells. Viral growth kinetics were monitored by measuring extracellular p24 antigen production. All 4 viruses demonstrated similar *in vitro* replication kinetics, suggesting that the L74I polymorphism does not impact virus growth (Fig. 1).

### Cabotegravir breakthrough experiments.

**FIG 2:**
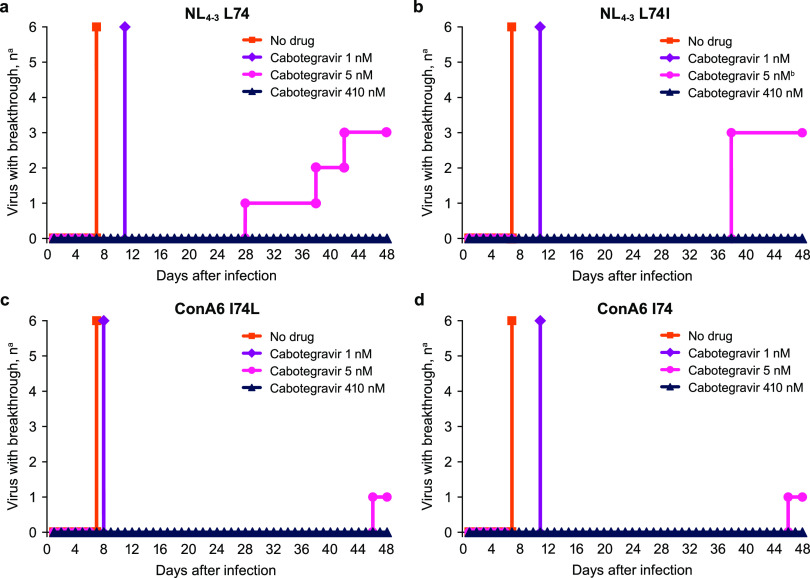
Viral breakthrough of (a) NL_4-3_ L74, (b) NL_4-3_ L74I, (c) ConA6 I74L, and (d) ConA6 I74 replication-competent viruses after treatment with no drug or increasing cabotegravir concentrations. The experiment was performed with 6 replicates per virus per treatment. Viral breakthrough was evaluated by visual inspection for syncytia formation. ConA6, consensus A6. ^a^Of 6 replicates. ^b^Q148R resistance mutation emerged in one of 3 breakthrough viruses.

Breakthrough experiments were performed to assess the impact of L74I and HIV-1 subtype on the ability of cabotegravir to suppress *in vitro* viral replication. Cultures were infected with replication-competent NL_4-3_ viruses with L74, L74I, or chimeric NL_4-3_ viruses containing ConA6 HIV-1 subtype A6 I74 or I74L integrase sequences. Infected cultures were treated with cabotegravir doses representing 2 × EC_50_ (1 nM), 10× EC_50_ (5 nM), and 1× protein-adjusted 90% effective concentration (410 nM); a no-drug sample was used as a control. Breakthrough, as assessed by syncytia formation, occurred for all 6 replicates of each virus from the no-drug control within 7 days and between 8 and 11 days after treatment with cabotegravir 1 nM (Fig. 2). Results were similar across HIV-1 subtypes B and A6, regardless of whether leucine or isoleucine was present at position 74. Treatment with 5 nM cabotegravir resulted in viral breakthrough in 3 of 6 replicates for NL_4-3_ viruses, occurring between Days 28 and 42 for NL_4-3_ viruses with L74 and on Day 38 for NL_4-3_ viruses with L74I. Viral breakthrough occurred in 1 replicate each on Day 46 for chimeric ConA6 HIV-1 subtype A6 strains with either I74 or I74L. Viral breakthrough did not occur with 410 nM cabotegravir for any virus within 56 days. Emergence of resistance mutations in breakthrough viruses was rare, with only 1 replicate of NL_4-3_ L74I containing a Q148R mutation with 5 nM cabotegravir. No other resistance mutations emerged at any amino acid position in any breakthrough virus after treatment with 1 or 5 nM cabotegravir. No Q148R mutations were observed when additional replicates were repeated in 3 independent experiments at 5 nM cabotegravir for ≥100 days, indicating that the occurrence of Q148R in combination with L74I was rare at low concentrations and not observed at 410 nM, a concentration of cabotegravir below the mean minimum observed plasma concentration at steady-state in LA treatment studies (2.97 μg/ml) (42).

## DISCUSSION

In this study, the effect of the L74I integrase polymorphism on susceptibility to cabotegravir was examined using NL_4-3_ HIV-1 subtype B viruses and chimeric NL_4-3_ viruses containing integrase sequences derived from a consensus HIV-1 subtype A6 sequence. Fold change in EC_50_ for both NL_4-3_ L74I and ConA6-containing I74L mutants compared with their parent viruses with L74 and I74 was near 1 for all viruses, indicating similar susceptibility to cabotegravir, regardless of whether L74I was present or not. The L74I polymorphism in combination with Q148R resulted in a 4-fold change in EC_50_ in viruses containing integrase genes from both HIV-1 subtypes; however, Q148R alone produced a 5-fold increase in EC_50_ in a previous study, suggesting that L74I likely did not predispose these viruses to resistance (41). Furthermore, frequency and timing of viral breakthrough, as assessed by syncytia formation, after *in vitro* cabotegravir inhibition at various cabotegravir concentrations was similar across viruses containing integrase genes from the 2 HIV-1 subtypes, regardless of leucine or isoleucine at amino acid 74, suggesting that having the L74I polymorphism at baseline does not predispose for selection of *in vitro* resistance to cabotegravir. In addition, the emergence of Q148R in combination with L74I was rare at low cabotegravir concentrations, with only 1 replicate containing a Q148R mutation with 5 nM cabotegravir after 48 days and no selection of Q148R occurring when additional replicates were repeated at 5 nM cabotegravir for ≥100 days. Therefore, presence of the baseline L74I integrase polymorphism does not impact the *in vitro* potency or durability of cabotegravir suppression.

A total of 54 participants who received cabotegravir LA + rilpivirine LA in the FLAIR study had the L74I polymorphism at baseline, 50 of whom had HIV-1 RNA <50 copies/mL at Week 48 and 3 of whom met CVF criteria on cabotegravir LA + rilpivirine LA (28). The remaining participant met CVF criteria upon reinitiating oral lead-in therapy after treatment with oral cabotegravir + rilpivirine was suspended because of a false-positive pregnancy test and never received LA dosing. The proportion of FLAIR study participants receiving LA therapy with HIV-1 RNA <50 copies/mL at Week 48 was similar with or without the L74I polymorphism at baseline (93% vs 94%). Thus, presence of the L74I polymorphism appeared not to be associated with decreased treatment efficacy in FLAIR. In a pooled analysis of 1,039 participants across the FLAIR, ATLAS, and ATLAS-2M phase III studies through Week 48, 13 (1.3%) participants had CVF, 8 of whom had the baseline integrase L74I polymorphism (38). However, a multivariable analysis found no statistically significant association between L74I and increased odds of CVF. The lack of association between L74I and CVF across cabotegravir LA + rilpivirine LA phase III studies is consistent with the findings from this analysis, which showed that L74I did not impact *in vitro* sensitivity to cabotegravir.

In addition to the participants with CVF included in this analysis, HIV-1 subtype was also re-evaluated in a larger population of participants from FLAIR. Of note, this analysis did not include participants from South Africa because consent was not obtained for further virology exploration. After re-evaluation of HIV-1 subtype, 40 participants in the FLAIR study were identified as having HIV-1 subtype A6, with the L74I polymorphism present in 36 participants (including the 3 participants with CVF) and the wild-type L74 position present in the other 4 participants (including 2 participants who were classified as having HIV-1 subtype A1 in the original analysis). In the 48-week pooled analysis of FLAIR, ATLAS, and ATLAS-2M, 106 (88.3%) of 120 participants with HIV-1 subtype A6/A1 also had the L74I polymorphism; of those 106 participants, 7 (6.6%) had CVF (38). One additional participant with CVF had L74I and HIV-1 subtype C. Since the 48-week analysis in ATLAS-2M, 1 participant with HIV-1 subtype B and the L74I polymorphism met CVF criteria; this individual had RT mutations K103N and Y181C but no INSTI RAMs at CVF (43). Thus, HIV-1 subtype A6/A1 in combination with L74I had an apparent association with CVF outcomes across the 3 phase III studies. These results contrast with the *in vitro* findings from the present analysis, which showed that L74I did not differentially impact the sensitivity or durability of cabotegravir for HIV-1 subtypes A6 or B and highlight the multifactorial nature of virologic failures.

In the pooled analysis of the FLAIR, ATLAS, and ATLAS-2M studies, the presence of factors in addition to the L74I polymorphism was evaluated for potential association with CVF outcomes (38). A multivariable logistic regression analysis found that increased odds of CVF were significantly associated with HIV-1 subtype A6/A1, baseline rilpivirine RAMs, Week 8 rilpivirine trough concentrations, and baseline body mass index. Participants with ≥2 of the significant baseline factors HIV-1 subtype A6/A1, body mass index ≥30 kg/m^2^, or baseline rilpivirine RAMs had a moderately increased risk of experiencing CVF compared with those who had 0 or 1 baseline factor. In addition to having the baseline L74I polymorphism and HIV-1 subtype A6, all 3 FLAIR study participants with CVF had a body mass index >30 kg/m^2^ (28). Plasma drug levels in these participants were also in the lowest quartile, although no history of maladministration or dosing complications were reported. Because only 1 out of 9 breakthrough viruses with L74I in HIV-1 subtype B selected for Q148R, it is unlikely that the low cabotegravir levels selected for Q148R in these FLAIR study participants who had HIV-1 subtype A6. Therefore, other demographic and/or pharmacokinetic factors besides the L74I polymorphism and HIV-1 subtype A6/A1 may have contributed to CVF during LA therapy.

In addition to the L74I baseline integrase polymorphism, treatment-emergent resistance mutations in integrase were found in the 3 FLAIR study participants with CVF: 1 with G140R and 2 with Q148R (28). In this *in vitro* study, NL_4-3_ mutants with L74I plus G140R and ConA6-containing mutants with I74 plus G140R were fully susceptible to cabotegravir. This observation contrasts with results from the FLAIR study in which HIV-1 subtype A6 virus with L74I and G140R mutations from the study participant with CVF demonstrated reduced susceptibility to cabotegravir by 6.7-fold versus the reference virus (28). This discrepancy may be due to additional integrase amino acid substitutions (K14R and L63V) in this participant and is being evaluated; however, it is unclear how these N-terminal domain polymorphisms would impact cabotegravir potency. By contrast, site-directed mutants with L74I and Q148R in NL_4-3_ viruses and I74 and Q148R in ConA6-containing viruses exhibited reduced susceptibility to cabotegravir *in vitro*, an observation that is consistent with the results from FLAIR (28), which demonstrated a 5.2- to 9.4-fold change in cabotegravir susceptibility for each HIV-1 subtype A6 virus from participants with CVF versus the reference virus. Given that L74I did not impact susceptibility to cabotegravir *in vitro*, it is likely that Q148R was the primary factor affecting cabotegravir resistance, which is a possibility being further investigated.

Treatment-emergent mutations in RT were also identified in the 3 participants with CVF from FLAIR: 1 with K101E, 1 with E138E/A/K/T, and 1 with E138K (29). Site-directed mutants with K101E showed *in vitro* resistance to rilpivirine, which is consistent with the results from FLAIR that showed a 2.63-fold change in rilpivirine susceptibility for the participant with CVF and K101E. In this *in vitro* study, E138A and E138T mutations in isolation were susceptible to rilpivirine, while E138K demonstrated *in vitro* resistance to rilpivirine. These observations contrast with those from the FLAIR study, in which the participant with E138E/A/K/T mixtures had a 7.1-fold change in rilpivirine susceptibility, and the participant with E138K alone was susceptible to rilpivirine. As the presence of E138K alone has been associated with decreased susceptibility to rilpivirine (44), the discrepancy observed between observations from this analysis and those from the FLAIR study are unclear. Similar to the observations for cabotegravir, rilpivirine susceptibility was comparable between HIV-1 subtypes A1 and B, in both the absence and presence of the RT RAMs observed in the participants with CVF in FLAIR.

This study has limitations. The vectors used to generate the chimeric ConA6 HIV-1 subtype A6 strains had an HIV-1 subtype B backbone. Because a consensus sequence for the subtype A6 integrase was used, the sequences of the site-directed mutant viruses generated for the cabotegravir experiments were not completely identical to those from viruses isolated from FLAIR participants. As the HIV-1 subtype reclassification was not known when the rilpivirine experiments were initiated, a true HIV-1 subtype A1 reference strain was selected to assess rilpivirine susceptibility instead of a subtype A6 reference virus as was done for cabotegravir. Comparison of the HIV-1 subtype A1 reference strain 92UG037 (GenBank accession number AB253428) containing the consensus RT sequence at baseline and failure from the 3 participants with CVF, but not the ConA6 integrase sequence, revealed 19 amino acid differences but only at positions not associated with NNRTI resistance (data not shown).

Results from this study suggest that factors other than the presence of L74I at baseline and integrase genes from HIV-1 subtype A6 may contribute to the low percentage of INSTI and NNRTI resistance in participants with CVF after cabotegravir LA + rilpivirine LA treatment. In addition to G140R and Q148R integrase mutations, other integrase polymorphisms present in viral sequences from FLAIR study participants with CVF may have impacted cabotegravir resistance. All observed RT mutations have been previously identified as being associated with resistance to rilpivirine except for E138T, which did not result in reduced rilpivirine susceptibility in isolation (44, 45). Given the development of RAMs in participants with CVF, it is important that selection of people living with HIV-1 for treatment with cabotegravir LA + rilpivirine LA aligns with the approved labeling indications, including lack of known or suspected resistance to either cabotegravir or rilpivirine (23). Overall, results from this study provide valuable knowledge for understanding cabotegravir and rilpivirine resistance after LA therapy.

## MATERIALS AND METHODS

### Study approval and consent.

The study protocol for FLAIR was reviewed and approved by an investigational center ethics committee or institutional review board in accordance with the International Conference on Harmonisation of Technical Requirements for Registration of Pharmaceuticals for Human Use Good Clinical Practice and the Declaration of Helsinki. Participants provided written informed consent before any study procedure was performed.

### HIV-1 subtype classification.

The updated classification of HIV-1 subtype in FLAIR study participants with CVF was performed using integrase reference sequences obtained from the 2018 version of the LANL HIV sequence database (http://www.hiv.lanl.gov/). The reference panel included 80 full-length genomes from noncirculating recombinant forms representing 14 HIV-1 subtypes, including A1 (*n* = 8), A2 (*n* = 1), A3 (*n* = 1), A4 (*n* = 1), A6 (*n* = 4), B (*n* = 15), C (*n* = 15), D (*n* = 10), F1 (*n* = 8), F2 (*n* = 2), G (*n* = 9), H (*n* = 2), J (*n* = 2), and K (*n* = 2). HIV-1 subtype was assigned based on Smith–Waterman similarity score for pairwise comparison between sample sequence and each reference sequence (46). This HIV-1 subtype analysis differed from that used by Monogram Biosciences (South San Francisco, CA) in the original FLAIR study analysis by the addition of integrase sequences to the algorithm that contained PR and RT sequences and use of an updated LANL reference panel with the inclusion of HIV-1 subtypes A3, A4, and A6 and removal of circulating recombinant forms and HIV-1 subtypes O and N.

### Proviral vectors.

NL_4-3_–based proviral pseudotyping vectors (NLCH, the parent molecular infectious clone kindly provided by the laboratory of Ron Swanstrom [University of North Carolina at Chapel Hill, Chapel Hill, NC], is a modification of HIV-1 NL_4-3_ [GenBank U26942] where flanking genomic sequences were removed) that contained a lethal mutation in the envelope open-reading frame and a firefly luciferase reporter in place of the nef open-reading frame were used to create HIV-1 mutants. A chimeric env- vector of HIV-1 with the integrase open-reading frame from subtype A6 was generated by replacing the NL_4-3_ integrase sequence with the consensus integrase sequence from the baseline sequences derived from the 3 FLAIR participants with CVF (ConA6); I74 was consensus in the ConA6 vector. Mutations (L74I, G140R, Q148R, and I74L) were introduced into the NL_4-3_ or ConA6 parental vectors by site-directed mutagenesis using the Q5 site-directed mutagenesis kit (New England Biolabs, Ipswich, MA). In addition, a full-length NL_4-3_ proviral vector that produces replicating virus was obtained from the National Institutes of Health AIDS Reagent Program (Division of AIDS, National Institute of Allergy and Infectious Diseases, National Institutes of Health, Bethesda, MD), and a chimeric NL_4-3_ ConA6 vector was constructed by replacing the NL_4-3_ integrase sequences with the ConA6 integrase sequences. Variants with or without I74L integrase mutations were constructed using site-directed mutagenesis.

### *In vitro* cabotegravir susceptibility assays.

Replication-defective pseudoviruses were produced by cotransfection of either the env- NL_4-3_–based or ConA6 vectors with a cytomegalovirus-expressed HIV-1 gp160 envelope vector into HEK 293T cells using PolyFect transfection reagent (Qiagen, Hilden, Germany). Viruses were harvested after 48 h and stored at −80°C.

U-373 cells stably expressing a β-galactosidase reporter, human CD4, and either HIV C-X-C chemokine receptor 4 or C-C chemokine receptor 5 coreceptors were treated with either media or a dose range of cabotegravir concentrations. Treated cells were infected with replication-defective NL_4-3_ or ConA6 pseudoviruses in 100-μL volume for 30 min at room temperature and incubated at 37°C for 3 days in 3 independent experiments. Luciferase activity was measured using a TopCount luminescence reader (Perkin Elmer, Waltham, MA) and used to calculate cabotegravir EC_50_. Raw luciferase activity data were reported as relative light units (RLU; luciferase/fusion component) expressed as percent of control using the following equation: percent of control = (RLU with drug/RLU with no drug) × 100. Values for EC_50_ were determined using the following nonlinear regression model available in Robosage software (GlaxoSmithKline, Brentford, UK): *y* = Vmax × {1 – [*x^n^*/(*K^n^* + *x^n^*)]} + *Y*2, where *y* is the response being inhibited, Vmax is the maximum rate of metabolism, *x* is the inhibitor concentration, *K* is the EC_50_ for the inhibition curve (i.e., *y *= 50% of Vmax when *x *=* K*), *n* is the Hill coefficient, and *Y*2 is baseline.

### *In vitro* rilpivirine susceptibility assays.

DNA fragments encoding PR/RT from the NL_4-3_ clone (HIV-1 subtype B) or the 92UG037 reference strain (HIV-1 subtype A1) with or without K101E, E138A, E138K, or E138T RT mutations were synthesized and ligated into replication-defective proviral vectors at Monogram Biosciences (47). A reference strain of HIV-1 subtype A1 was used to assess rilpivirine susceptibility because the reclassification from HIV-1 subtype A1 to A6 was not known when the rilpivirine experiments were initiated. Resulting recombinant viruses were subjected to *in vitro* rilpivirine susceptibility testing using PhenoSense technology (Monogram Biosciences) to measure fold change in EC_50_ in 3 independent experiments.

### Virus growth and breakthrough with cabotegravir.

Full-length NL_4-3_ proviral vectors and chimeric ConA6 integrase gene-containing proviral vectors in the NL_4-3_ backbone, with or without L74I integrase mutations, were used to prepare virus stocks as previously described (48). MT-2 cells were infected with equivalent p24 values, and replication kinetics were evaluated by quantifying extracellular HIV-1 p24 antigen concentrations using the HIV p24 high sensitivity AlphaLISA Detection Kit (Perkin Elmer). For the cabotegravir breakthrough experiments, MT-2 cells were infected with each virus at a multiplicity of infection of 0.005 or 0.001 in replicates of 6. After 16 h, cultures were treated with 0.2% dimethyl sulfoxide in either media, 2 × EC_50_ (1 nM), 10× EC_50_ (5 nM), or 1 × protein-adjusted 90% effective concentration (410 nM) of cabotegravir. Cells were refreshed by removing half the volume of cell culture media and adding an equal volume of media every 3 to 4 days while maintaining the same concentration of cabotegravir. Infection with NL_4-3_–based HIV-1 isolates is associated with syncytia formation in MT-2 cells (49). Cultures were evaluated for viral breakthrough by visual inspection for syncytia for 56 days, after which the integrase genes of breakthrough viruses were amplified via PCR from cDNA with forward primer 5-GCATTAGGAATCATTCAAGCACAACCAGA and reverse primer 5-GACCCAAATGCCAGTCTCTTTCTCCTGT. The 1,207-base pair product was run on an E-Gel agarose gel (0.8%; Invitrogen, Waltham, MA) and purified with the MinElute PCR purification kit (Qiagen). Samples were sequenced and analyzed using SeqMan Pro (DNASTAR, Madison, WI).

### Statistical analysis.

A log_10_ transformation was applied to the EC_50_ values before the analysis to make variances more homogeneous. A separate analysis was performed for each viral vector. Experiments were performed across several days, and we used a linear mixed-effects model with mutation as a fixed effect and day as a random effect to account for potential differences between experimental days. Dunnett’s multiple comparison adjustment was used to compare all mutants with the respective wild-type virus. Estimated differences and confidence intervals were back-transformed to the original scale, resulting in fold changes and their respective confidence intervals. Data were analyzed using PROC MIXED in SAS 9.4 (SAS Institute, Cary, NC).

### Data availability.

Anonymized individual participant data and study documents can be requested for further research from www.clinicalstudydatarequest.com.
